# Associations between muscle dysmorphia-related body image concerns, physical activity, and body composition: a cross-sectional study among young Asian adults in Malaysia

**DOI:** 10.1186/s40101-026-00427-9

**Published:** 2026-05-19

**Authors:** Yee-How Say, Shi-Hui Cheng, Nur Fadhilah Syuhada Nazaruddin, Jingruo Wang, Wen Yi Goh, Fei Ling Yap, Meng-Che Tsai

**Affiliations:** 1 Department of Biomedical Sciences, Jeffrey Cheah Sunway Medical School, Faculty of Medical and Life Sciences, Sunway University, 5 Jalan Universiti, Bandar Sunway, Subang Jaya, Selangor 47500 Malaysia; 2 School of Biosciences, Faculty of Science and Engineering, University of Nottingham Malaysia, Jalan Broga, Semenyih, Selangor 43500 Malaysia; 3 Department of Biomedical Sciences, Faculty of Science and Engineering, University of Nottingham Malaysia, Jalan Broga, Semenyih, Selangor 43500 Malaysia; 4 Department of Pediatrics, College of Medicine, National Cheng Kung University Hospital, National Cheng Kung University, Tainan, Taiwan; 5 Department of Medical Humanities and Social Medicine, College of Medicine, National Cheng Kung University, Tainan, Taiwan

**Keywords:** Muscle dysmorphia, Body image, Physical activity, Body composition, Young adults

## Abstract

**Background:**

Muscle dysmorphia (MD) is a body image disturbance characterized by an obsessive drive for muscularity, increasingly observed in non-Western populations. However, limited data exist on its associations with physical activity (PA) and body composition in Asian contexts. This study examined the relationships between MD-related body image concerns, PA levels, and anthropometric/body composition characteristics among young Asian adults in Malaysia.

**Methods:**

A cross-sectional study involving 513 university students in Malaysia (207 males, 306 females; median age 21 ± 2 years) was conducted. Participants completed validated questionnaires assessing MD-related concerns—Drive for Leanness Scale (DLS), Drive for Muscularity Scale (DMS), MD Disorder Inventory (MDDI), Muscularity-Oriented Eating Test (MOET)—and weight control behaviors. PA was assessed using the IPAQ-SF, and body composition was measured via bioimpedance. Sex differences, correlations, and multiple regression analyses were performed.

**Results:**

Males reported significantly higher DMS and MDDI scores, greater skeletal muscle, and higher PA levels (*p* < 0.001), while females had higher adiposity measures. PA showed positive associations with all MD-related body image concerns, including DMS (β = 0.149), MDDI (β = 0.188), and MOET (β = 0.107). Regression models also identified BMI, waist-to-height ratio, subcutaneous fat, financial adequacy, and life satisfaction as significant associated factors. Participants in higher PA categories reported more severe MD-related concerns.

**Conclusion:**

Overall, the findings indicate that while PA and body composition are statistically associated with MD-related concerns, these relationships are relatively weak, suggesting that objective physical characteristics play a limited role. This underscores the importance of considering psychological and sociocultural factors when examining MD among young Asian adults.

**Supplementary Information:**

The online version contains supplementary material available at 10.1186/s40101-026-00427-9.

## Background

Body image is a complex, multidimensional construct encompassing perceptions, thoughts, and feelings about one’s physical appearance, influenced by biological, psychological, and sociocultural factors [1]. While traditional research has largely focused on thinness ideals—particularly among females—emerging evidence indicates that muscularity has become a dominant body ideal among males [2]. Muscle dysmorphia (MD), a subtype of body dysmorphic disorder, involves a pathological preoccupation with one’s muscularity and body size, often leading to compulsive exercise, disordered eating, and distress over perceived inadequacy despite having a muscular physique [2, 3]. Though first described in Western populations, this phenomenon is increasingly observed in non-Western contexts, including Asian societies, where globalization and social media have contributed to the internalization of muscular body ideals [4, 5].

Young adults, particularly those in university settings, are especially vulnerable to developing body image concerns. This developmental stage is marked by heightened self-consciousness, identity exploration, and susceptibility to social comparison [6]. University students are frequently exposed to media portrayals of idealized bodies and peer influences that promote certain appearance standards [7–9]. While regular physical activity (PA) is associated with improved physical and mental health, its relationship with body image is more nuanced. Some individuals engage in exercise not for health, but to attain socially idealized physiques, which may be symptomatic of MD [10–13]. Simultaneously, objective body composition characteristics—such as body fat, waist-to-hip ratio, and skeletal muscle mass—play a key role in shaping body image but are rarely examined alongside psychological variables.

Despite the growing visibility of muscularity-related concerns, few studies have investigated how these concerns relate to both PA and body composition within Asian populations [4, 14]. In Malaysia, Western ideals of muscularity coexist with traditional cultural values, potentially amplifying appearance-related pressures [4]. However, empirical data on muscle dysmorphia in Malaysian or broader Asian contexts remain scarce [4]. Existing literature has largely focused on body dissatisfaction and eating disorders among females, with limited attention to muscularity concerns among males and the role of PA as a behavioral correlate [15].

There is a need to understand how body image disturbances manifest among Malaysian youth and how these concerns relate to health behaviors and physiological markers. Exploring these relationships may provide insights into early identification of at-risk individuals and inform preventive interventions tailored to local sociocultural contexts. Moreover, examining sex-specific patterns can guide the development of gender-sensitive health promotion strategies, especially within academic settings where such issues often emerge.

The present study aimed to examine the associations between MD-related body image concerns, PA levels, and anthropometric/body composition characteristics among young Asian adults in Malaysia. Specifically, this study sought to (1) compare MD-related body image concern scores, PA levels, and body composition by sex; (2) assess the relationships between MD-related body image concerns, PA, and anthropometric/body composition indicators; and (3) identify associated factors of MD-related body image concerns, adjusting for relevant sociodemographic and psychosocial factors.

## Methods

### Study design and participants

This study was reported in accordance with the Strengthening the Reporting of Observational Studies in Epidemiology (STROBE) guidelines for cross-sectional studies. A completed STROBE checklist is provided in Supplementary Table S1. Participants were recruited through purposive and convenience sampling methods via campus-wide emails, social media advertisements, and student groups at Sunway University Malaysia and Nottingham University Malaysia, from May 2024 to March 2025. Inclusion criteria required participants to be full-time university or college students aged 18–25 years, willing to provide informed consent, and self-identify as having Asian descent. Exclusion criteria included individuals with self-reported chronic illnesses, eating disorders, or physical disabilities that could affect body composition, PA, or psychological well-being. Using the Raosoft sample size online calculator (http://www.raosoft.com/samplesize.html), a minimum sample size of 380 is needed to achieve a 5% margin of error, 95% confidence level, a combined population size of both institutions of 30,000, and a 50% response distribution.

This study was conducted following ethical research principles as outlined in the Declaration of Helsinki and received ethical approvals from the Sunway University Research Ethics Committee (SUREC) (Approval ID: SUREC 2024/029) and the University of Nottingham Malaysia Faculty of Science and Engineering Research Integrity and Research Ethics Committee (Approval ID: CSH210624). All participants provided informed written consent in the online Google Form before completing the survey, and their responses were anonymous and confidential. All questions in the Google Form were set as compulsory, and full completion is required before clicking the “submit” button. Therefore, the participation rate was considered as 100%.

### Measures

#### Sociodemographic and psychosocial variables

Participants provided information on age, sex, ethnicity, educational level, employment status, perceived financial adequacy, perceived health status, perceived quality of life, food insecurity, and unmet healthcare needs. Psychological well-being was assessed using the WHO-5 Well-Being Index, a validated five-item scale measuring subjective emotional well-being over the past 2 weeks [16].

#### Drive for Leanness Scale (DLS)

The DLS [17] is a six-item measure of men and women’s desire to have limited body fat and toned, healthy muscles. The severity of each symptom is reported by the participants, with items rated on a 5-point Likert-type scale ranging from 1 (always) to 5 (never). Total scores could range from 6 to 30, with higher scores reflecting a stronger drive for leanness. Cronbach’s α was good (0.79 and 0.83 for men and women, respectively) [17].

#### Drive for Muscularity Scale (DMS)

The DMS [18] is a 15-item measure used to evaluate drive for muscularity. Two subscales measuring attitudes and behaviors related to muscularity comprise the measure, which produces a total score. The severity of each symptom is reported by the participants, with items rated on a 5-point Likert-type scale ranging from 1 (always) to 5 (never). Higher scores indicate a stronger desire for muscularity as the items were reverse-coded to have the same directionality as other study measures. Cronbach’s α was good, ranging from 0.81 to 0.87 for both total and subscale scores [19].

#### Muscle Dysmorphia Disorder Inventory (MDDI)

The MDDI [20] is a 13-item questionnaire to evaluate the presence of MD. The MDDI is divided into three subscales: drive for size (5 items), appearance intolerance (4 items), and functional impairment (4 items). The severity of each symptom is reported by the participants, with items rated on a 5-point Likert-type scale ranging from 1 (always) to 5 (never). A total score can be derived by the sum of all items or all subscales. Higher total scores indicate more symptoms of MD. MDDI has good internal consistency (Cronbach’s α = 0.81) and excellent retest reliability (*r* = 0.87) [20].

#### Muscularity-Oriented Eating Test (MOET)

The MOET [21] is a 15-item questionnaire to detect muscularity focused on muscularity-oriented eating disorders. The severity of each symptom is reported by the participants, with items rated on a 5-point Likert-type scale ranging from 1 (always) to 5 (never). High scores from the scale and its subfactors indicate a high level of muscularity-oriented eating disorder. Strong internal consistency was found in two separate studies (study 1: omega = 0.93; study 2: omega = 0.92) and a high test–retest correlation (rs = 0.75) [21].

#### Weight Loss Experience Questionnaire

Four self-designed questions were designed to assess whether someone has engaged in potentially unhealthy behaviors related to weight management within the past 3 months, based on a systematic review and meta-analysis [22]. These included (1) have you tried dieting to lose or maintain weight?; (2) Have you tried medication to lose or maintain weight?; (3) Have you tried to induce vomiting to lose or maintain weight?; and (4) Have you tried excessive exercise in the past 3 months to lose or maintain weight? Options and scores were 1 = no; 2 = yes. The sum of scores was calculated, and higher total scores indicate a higher prevalence of weight loss experience.

#### PA assessment

PA was assessed using the short form of the International PA Questionnaire (IPAQ-SF), which measures self-reported frequency and duration of vigorous, moderate, and walking activity over the past 7 days [23]. Participants were categorized into low, moderate, and high activity groups based on the official IPAQ scoring protocol, expressed in MET-minutes per week. The total PA level (MET-mins/week) is then classified into three classes: low (< 600), moderate (600–2999.99), and high (≥ 3000) PA [23].

#### Anthropometric and body composition measurements

Height was measured using a wall-mounted stadiometer. Waist and hip circumferences were measured using a stretch-resistant tape that provided a constant 100 g tension, at the midpoint between the lower margin of the least palpable rib and the top of the iliac crest and around the widest portion of the buttocks, respectively [24]. The waist-hip ratio (WHR) and waist-to-height ratio (WHtR) were calculated by dividing waist circumference (WC) by hip circumference and height, respectively. A bioimpedance body composition scale (Omron HBF-375) was used to determine weight, body mass index (BMI; kg/m^2^), total body fat (TBF; %), visceral fat level (VFL; %), subcutaneous fat (SF; %), skeletal muscle percentage (SM; %) and resting metabolism rate (RM; kcal). The cut-off points for overweight, obesity, high TBF, high VFL, high SM, high WC, high WHR and high WHtR are ≥ 23 kg/m^2^ [25]; ≥ 27.5 kg/m^2^ [25]; 20% (men) or 30% (women) [26]; 10% [26]; 35.8% (men) or 28.0% (women) [26]; 90 cm (men) or 80 cm (women) [25]; 0.90 (men) or 0.85 (women) [24]; and 0.50 [27], respectively.

### Statistical analysis

Data were analysed using IBM SPSS Statistics for Windows 26.0 (IBM Corp., Armonk, NY, USA). Data completeness was evaluated prior to analysis. The proportion of missing data was low, with fewer than 5% of observations missing for any individual variable. Participants with missing data on primary outcome variables (muscle dysmorphia–related body image measures) were excluded from the relevant analyses using a complete-case approach. For covariates, analyses were conducted using all available data for each model. Examination of missingness patterns did not suggest systematic differences between participants with complete and incomplete data. Given the minimal extent of missing data and the absence of evidence for non-random missingness, imputation methods were not applied. All analyses were therefore based on available complete data, in line with STROBE recommendations for transparent reporting. Descriptive statistics were used to summarize participant characteristics. The conformity of the continuous variables to normal distribution was determined by the Kolmogorov–Smirnov test, where *p* > 0.05 indicates normally distributed data. As the continuous variables were not normally distributed, they were reported as medians with interquartile ranges (IQRs), while categorical variables were presented as frequencies and percentages. Between-group comparisons by sex were performed using Chi-square tests for categorical variables and Mann–Whitney *U* tests for non-normally distributed continuous variables. Examination of the relationships between PA, MD-related body image scores, and body composition indices were determined by rank-based partial correlation test controlling for confounding socio-demographic factors, i.e. sex, ethnicity, current education status, work status, financial adequacy, health perception, life perception, food insecurity, health need, and WHO-5 well-being score was performed as follows: (1) rank-transforming the PA levels, MD-related body image concern scores, and anthropometric/body composition measurements; (2) regress out prespecified covariates confounding factors by linear regression (rank-transformed scores as dependents and covariates as independents, and saving the new variables as unstandardized residuals; and (3) Pearson’s bivariate correlation analysis of the unstandardized residuals, with 95% confidence interval and bias adjustment. In the interpretation of the correlation coefficient, it was determined as a “very weak correlation, if < 0.2”, a “weak correlation between 0.2 and 0.4”, a “moderate correlation between 0.4 and 0.6”, a “high correlation between 0.6 and 0.8”, and “ > 0.8 very high correlation”. Multiple linear regression analysis was performed with the following associated factors—constant: socio-demographic factors and anthropometric/body composition data; dependent variable: MD-related body concern scores. The following assumptions were used in the multiple linear regression analysis: linear relationship between the dependent variable and each independent variable, independence and normality of errors, homoscedasticity, no multicollinearity, no outliers, and sufficient sample size. Sensitivity analyses excluding extreme PA values and using alternative anthropometric specifications yielded comparable results, supporting the robustness of the main findings. Statistical significance was set at *p* < 0.05.

## Results

### Sociodemographic and lifestyle characteristics

A total of 513 young Asian adults participated in the study, comprising 207 males and 306 females, with a median age of 21 ± 2 years across both sexes. The sample was predominantly Chinese, followed by Malay/Bumiputra, non-Malaysian Asians, and Indians. A significant difference in ethnic distribution was observed between sexes (*p* = 0.036), with males more frequently identifying as non-Malaysian Asian and females as Chinese (Table 1). While the majority of participants were undergraduate students, males were more likely to report part-time work than females (28.0% vs. 20.3%, *p* = 0.042). No sex differences were found in perceived financial adequacy (*p* = 0.244) or food insecurity (*p* = 0.461). However, males were more likely to rate themselves as “very healthy” compared to females, who more often reported being “somewhat unhealthy” (*p* = 0.008). There were no significant sex differences in perceived life satisfaction (*p* = 0.178), health needs (*p* = 0.051), or WHO-5 well-being categories (*p* = 0.448) (Table 1).

**Table 1:** Baseline sociodemographic, lifestyle, and anthropometric/body composition characteristics of overall participant and according to sex

Variables	Male (*n* = 207)	Female (*n* = 306)	Total (*N* = 513)
Median age ± interquartile range (IQR)	21 ± 2	21 ± 2	21 ± 2
Ethnicity			
Malay/Bumiputra	22 (10.6)	53 (17.3)	75 (14.6)
Chinese	131 (63.3)	199 (65.0)	330 (64.3)
Indian	10 (4.8)	13 (4.2)	23 (4.5)
Non-Malaysian Asian	44 (21.3)	41 (13.4)	85 (16.6)
*χ*^2^; *p*	8.535; 0.036^*^		
Current education status			
Pre-university	52 (25.1)	63 (20.6)	115 (22.4)
Undergraduate	150 (72.5)	223 (72.9)	373 (72.7)
Postgraduate	5 (2.4)	20 (6.5)	25 (4.9)
*χ*^2^; *p*	5.436; 0.066		
Work status			
No	149 (72.0)	244 (79.7)	393 (76.6)
Yes (part-time)	58 (28.0)	62 (20.3)	120 (23.4)
*χ﻿*^2^; *p*	4.147; 0.042^*^		
Financial adequacy			
Very inadequate	8 (3.9)	13 (4.2)	21 (4.1)
Somewhat inadequate	58 (28.0)	99 (32.4)	157 (30.6)
Fairly adequate	114 (55.1)	170 (55.6)	284 (55.4)
Very adequate	27 (13.0)	24 (7.8)	51 (9.9)
*χ*^2^; *p*	4.166; 0.244		
Do you feel healthy?			
Very unhealthy	10 (4.8)	12 (3.9)	22 (4.3)
Somewhat unhealthy	59 (28.5)	123 (40.2)	182 (35.5)
Fairly healthy	111 (53.6)	152 (49.7)	263 (51.3)
Very healthy	27 (13.0)	19 (6.2)	46 (9.0)
*χ﻿*^2^; *p*	11.805; 0.008^**^		
Do you think life is good?			
Not beautiful at all	6 (2.9)	7 (2.3)	13 (2.5)
Not beautiful	18 (8.7)	20 (6.5)	38 (7.4)
Fairly beautiful	137 (66.2)	230 (75.2)	367 (71.5)
Very beautiful	46 (22.2)	49 (16.0)	95 (18.5)
*χ﻿*^2^; *p*	4.922; 0.178		
Food insecurity			
Very worried	12 (5.8)	13 (4.2)	25 (4.9)
Little worried	44 (21.3)	78 (25.5)	122 (23.8)
Not worried	71 (34.3)	112 (36.6)	183 (35.7)
Not worried at all	80 (38.6)	103 (33.7)	183 (35.7)
*χ﻿*^2^; *p*	2.583; 0.461		
Felt the need to see a doctor, but did not go			
No	104 (50.2)	127 (41.5)	231 (45.0)
Yes	103 (49.8)	179 (58.5)	282 (55.0)
*χ*^2^; *p*	3.809; 0.051		
WHO-5 Well-being Category			
Poor	48 (23.2)	80 (26.1)	128 (25.0)
Good	159 (76.8)	226 (73.9)	385 (75.0)
*χ*﻿^2^; *p*	0.576; 0.448		
PA level			
Low	34 (16.4)	99 (32.4)	133 (25.9)
Moderate	76 (36.7)	145 (47.4)	221 (43.1)
High	97 (46.9)	62 (20.3)	159 (31.0)
*χ﻿*^2^; *p*	43.530; < 0.001^**^		
WC class			
Normal	162 (81.8)	217 (78.9)	379 (80.1)
High	36 (18.2)	58 (21.1)	94 (19.9)
*χ﻿*^2^; *p*	0.612; 0.434		
WHR class			
Normal	176 (88.9)	199 (72.4)	375 (79.3)
High	22 (11.1)	76 (27.6)	98 (20.7)
*χ﻿*^2^; *p*	19.138; < 0.001^**^		
WHtR class			
Normal	143 (72.2)	222 (80.7)	365 (77.2)
High	55 (27.8)	53 (19.3)	108 (22.8)
*χ﻿*^2^; *p*	4.726; 0.03*		
BMI category			
Normal	116 (56.0)	229 (74.8)	345 (67.3)
Overweight	69 (33.3)	49 (16.0)	118 (23.0)
Obese	22 (10.6)	28 (9.2)	50 (9.7)
*χ﻿*^2^; *p*	22.868; < 0.001^**^		
TBF class			
Normal	117 (59.4)	195 (71.2)	312 (66.2)
High	80 (40.6)	79 (28.8)	159 (33.8)
*χ*^2^; *p*	7.108; 0.008^**^		
VFL class			
Normal	168 (85.3)	260 (95.2)	428 (91.1)
High	29 (14.7)	13 (4.8)	42 (8.9)
*χ﻿*^2^; *p*	13.946; < 0.001^**^		
SM class			
Normal	105 (53.3)	174 (63.5)	279 (59.2)
High	92 (46.7)	100 (36.5)	192 (40.8)
*χ*^2^; *p*	4.942; 0.026^*^		

*WC* waist circumference, *WHR* waist-hip ratio, *WHtR* waist-height ratio, *TBF* total body fat, *VFL* visceral fat level, *BMI* body mass index, *SM* skeletal muscle. Numbers are frequencies and percentages within sex (in parentheses). *χ*^*2*^ *p* values by Pearson’s chi-square test, significant at ^*^p < 0.05 or ^**^p < 0.01

### PA levels and body composition by sex

PA levels differed significantly by sex, with males more likely to engage in high levels of PA (46.9% *vs*. 20.3%) and females more likely to be in the low PA category (32.4% *vs*. 16.4%; *p* < 0.001) (Table 1). Males also exhibited a higher proportion of elevated SM (*p* = 0.026), while females had significantly higher TBF (*p* = 0.008), VFL (*p* < 0.001), WHR (*p* < 0.001), and WHtR (*p* = 0.030). BMI categories also differed by sex (*p* < 0.001), with males more likely to be overweight or obese and females more often within the normal range. There was no significant sex difference in WC classification (*p* = 0.434).

### Sex differences in body image concerns

Internal consistency of the self-designed Weight Loss Experience Questionnaire assessed using McDonald’s omega demonstrated moderate reliability in the present sample (ω = 0.55). Comparisons of MD-related body image concerns showed that males scored significantly higher than females on the DMS (*p* < 0.001) and MDDI (*p* < 0.001) (Table 2). No significant sex differences were found in DLS scores (*p* = 0.067) or weight loss scores (*p* = 0.114). These results suggest that muscularity-oriented concerns are more pronounced among males, in parallel with their higher PA levels and greater SM (Table 1).

**Table 2:** Differences in muscle dysmorphia-related body image concerns, physical activity levels, and anthropometric/body composition between sexes

	Male (*n* = 207)	Female (*n* = 306)	*p*
DLS score	21.00 ± 7.00	21.00 ± 7.00	0.067
DMS score	42.00 ± 17.00	30.00 ± 19.00	< 0.001^**^
MDDI score	25.00 ± 12.00	21.50 ± 10.00	< 0.001^**^
MOET score	22.00 ± 16.00	20.00 ± 17.00	0.185
Weight loss score	5.00 ± 1.00	5.00 ± 1.00	0.114
Total PA level	2862.00 ± 3522.00	1209.60 ± 2274.00	< 0.001^**^
WC	79.00 ± 13.63	71.00 ± 12.74	< 0.001^**^
WHR	0.83 ± 0.09	0.80 ± 0.10	< 0.001^**^
WHtR	0.46 ± 0.08	0.44 ± 0.07	0.004^**^
TBF	18.00 ± 9.5	26.90 ± 6.83	< 0.001^**^
VFL	5.00 ± 5.00	2.00 ± 2.62	< 0.001^**^
RM	1568.00 ± 251.00	1172.50 ± 195.25	< 0.001^**^
BMI	22.15 ± 5.22	20.42 ± 4.21	< 0.001^**^
SF	12.80 ± 7.50	22.15 ± 5.67	< 0.001^**^
SM	35.40 ± 4.10	27.4 ± 2.62	< 0.001^**^

*DLS *Drive for Leanness Scale,* DMS *Drive for Muscularity Scale,* MDDI *Muscle Dysmorphia Disorder Inventory,* MOET* Muscularity-Oriented Eating Test,* WC* waist circumference, *WHR *waist-hip ratio, *WHtR *waist-height ratio, *TBF* total body fat, *VFL* visceral fat level, *BMI* body mass index, *SM* skeletal muscle. Values are median ± interquartile range. *p* values by Mann–Whitney *U* test, significant at ^*^p < 0.05 or ^**^p < 0.01

### Associations between PA, MD-related body image, and body composition

Table 3 shows partial correlations between PA, MD-related body image concerns, and body composition, adjusting for demographic and psychosocial factors. Total PA was weakly and positively correlated with WC, WHtR, VFL, and BMI, though not all were statistically significant. PA was significantly very weakly correlated with all MD-related body image scores, but not anthropometrics/body composition. MD-related concerns, namely MDDI, MOET, and weight loss score, showed very weak to weak positive correlations with central adiposity measures (WC, WHtR, VFL), BMI, and RM. SM was negatively associated with body image concerns, particularly MOET and weight loss motivation, indicating that higher muscle mass may relate to fewer dysmorphic concerns. All MD-related body image scores were moderately and highly interrelated, with the highest correlation between DMS and MDDI scores (*r* = 0.676, *p* < 0.001), reflecting cognitive-behavioral patterns in body image concerns.

**Table 3:** Correlation between physical activity levels, muscle dysmorphia-related body image concern scores, and anthropometric/body composition measurements

		Total PA level	DLS score	DMS score	MDDI score	MOET score	Weight loss score
WC	*r*	0.081	0.002	0.021	0.154	0.207	0.279
	*p*	0.077	0.961	0.655	0.001^**^	< 0.001^**^	< 0.001^**^
	95% CI (upper, lower)	− 0.009, 0.170	− 0.088, 0.092	− 0.070, 0.111	0.065, 0.241	0.119, 0.292	0.194, 0.360
WHR	*r*	0.024	0.063	0.056	0.117	0.204	0.159
	*p*	0.610	0.173	0.225	0.011^**^	< 0.001^**^	0.001^**^
	95% CI (upper, lower)	− 0.067, 0.113	− 0.028, 0.152	− 0.034, 0.145	0.027, 0.205	0.116, 0.288	0.069, 0.245
WHtR	*r*	0.074	0.028	0.029	0.152	0.228	0.313
	*p*	0.110	0.545	0.533	0.001^**^	< 0.001^**^	< 0.001^**^
	95% CI (upper, lower)	− 0.017, 0.163	− 0.062, 0.118	− 0.062, 0.119	0.063, 0.239	0.141, 0.312	0.229, 0.391
TBF	*r*	0.042	0.002	− 0.025	0.093	0.160	0.273
	*p*	0.366	0.972	0.590	0.044^*^	< 0.001^**^	< 0.001^**^
	95% CI (upper, lower)	− 0.049, 0.132	− 0.089, 0.092	− 0.115, 0.066	0.002, 0.182	0.071, 0.247	0.187, 0.354
VFL	*r*	0.065	− 0.033	0.003	0.119	0.179	0.344
	*p*	0.161	0.470	0.945	0.010^*^	< 0.001^**^	< 0.001^**^
	95% CI (upper, lower)	− 0.026, 0.154	− 0.123, 0.057	− 0.087, 0.094	0.029, 0.208	0.089, 0.265	0.261, 0.421
RM	*r*	0.066	− 0.044	0.023	0.125	0.132	0.278
	*p*	0.151	0.344	0.615	0.007^**^	< 0.001^**^	< 0.001^**^
	95% CI (upper, lower)	− 0.024, 0.156	− 0.134, 0.047	− 0.067, 0.113	0.034, 0.212	0.042, 0.220	0.192, 0.359
BMI	*r*	0.056	− 0.009	− 0.007	0.094	0.160	0.357
	*p*	0.204	0.835	0.871	0.032*	< 0.001**	< 0.001**
	95% CI (upper, lower)	− 0.031, 0.142	− 0.096, 0.077	− 0.094, 0.079	0.008, 0.179	0.075, 0.243	0.279, 0.430
SF	*r*	0.072	0.000	− 0.029	0.096	0.153	0.258
	*p*	0.118	0.997	0.534	0.037^*^	0.001^**^	< 0.001^**^
	95% CI (upper, lower)	− 0.019, 0.162	− 0.091, 0.091	− 0.119, 0.062	0.006, 0.186	0.063, 0.240	0.171, 0.341
SM	*r*	− 0.036	− 0.005	0.031	− 0.081	− 0.124	−0.212
	*p*	0.432	0.906	0.500	0.079	0.007^*^	< 0.001^**^
	95% CI (upper, lower)	− 0.126, 0.054	− 0.096, 0.085	− 0.059, 0.121	− 0.170, 0.009	− 0.212, − 0.034	− 0.297, − 0.124
Total PA level	*r*	1.000	0.120	0.176	0.162	0.187	0.211
	*p*	–	0.007^**^	0.001^**^	< 0.001^**^	< 0.001^**^	< 0.001^**^
	95% CI (upper, lower)	–	0.033, 0.204	0.091, 0.258	0.076, 0.245	0.102, 0.269	0.124, 0.290
DLS score	*r*		1.000	0.562	0.412	0.325	0.236
	*p*		–	< 0.001^**^	< 0.001^**^	< 0.001^**^	< 0.001^**^
	95% CI (upper, lower)			0.500, 0.619	0.337, 0.481	0.245, 0.400	0.152, 0.316
DMS score	*r*		0.562	1.000	0.677	0.479	0.237
	*p*		< 0.001^**^	–	< 0.001^**^	< 0.001^**^	< 0.001^**^
	95% CI (upper, lower)		0.500, 0.619		0.627, 0.721	0.409, 0.542	0.153, 0.317
MDDI score	*r*		0.412	0.677	1.000	0.661	0.324
	*p*		< 0.001^**^	< 0.001^**^	-	< 0.001^**^	< 0.001^**^
	95% CI (upper, lower)		0.245, 0.400	0.627, 0.721		0.609, 0.707	0.244, 0.399
MOET score	*r*		0.325	0.479	0.661	1.000	0.437
	*p*		< 0.001^**^	< 0.001^**^	< 0.001^**^	–	< 0.001^**^
	95% CI (upper, lower)		0.245, 0.400	0.409, 0.542	0.609, 0.707		0.364, 0.504

*DLS* Drive for Leanness Scale, *DMS* Drive for Muscularity Scale, *MDDI* Muscle Dysmorphia Disorder Inventory, *MOET* Muscularity-Oriented Eating Test, *WC* waist circumference, *WHR* waist-hip ratio, *WHtR* waist-height ratio, *TBF* total body fat, *VFL* visceral fat level, *BMI* body mass index, *SM* skeletal muscle. Partial correlation analysis, controlling for: sex, ethnicity, current education status, work status, financial adequacy, health perception, perceived life satisfaction, food insecurity, health need, and WHO-5 well-being score. *p*-vales are significant at ^*^p < 0.05 or ^**^p < 0.01

### Associated factors of MD-related body image concern scores

Multiple linear regression analyses identified total PA level as a significant associated factor of all MD-related body image outcomes. Higher PA levels predicted elevated DLS (β = 0.145, *p* = 0.005), DMS (β = 0.124, *p* = 0.009), MDDI (β = 0.172, *p* = 0.001), MOET (β = 0.100, *p* = 0.044), and weight loss scores (β = 0.175, *p* < 0.001) (Table 4). In addition, higher central adiposity–WHtR significantly predicted higher MDDI (β = 0.148, *p* = 0.049), MOET (β = 0.157, *p* = 0.038), and weight loss scores (β = 0.255, *p* = 0.001), while WHR significantly predicted higher MOET (β = 0.192, *p* = 0.005). SF was a negative associated factor for DLS (β = –0.378, *p* = 0.021). Psychosocial factors also played a role. Lower financial adequacy significantly predicted higher MDDI (*p* = 0.049) and MOET (*p* = 0.013) scores, and lower life satisfaction was significantly associated with elevated scores for DMS (*p* = 0.025) and MDDI (*p* = 0.034). Lower food insecurity significantly predicted higher MOET (*p* = 0.038). The models accounted for 6.4% to 17.9% of the variance in body image concern scores, with the highest explanatory power observed for the DMS model (adjusted *R*^2^ = 14.6%).

**Table 4:** Multiple linear regression analysis predicting muscle dysmorphia-related body image concern scores

Variable	*B*	β	*t*	*p*	95%CI	
					Lower	Upper
DLS score						
Sex	− 0.16	− 0.015	− 0.142	0.887	− 2.383	2.062
Ethnicity	0.234	0.039	0.786	0.432	− 0.351	0.818
Financial adequacy	− 0.404	− 0.053	− 1.079	0.281	− 1.141	0.332
Health perception	0.027	0.003	0.068	0.946	− 0.747	0.8
Life perception	−0.678	−0.073	−1.39	0.165	− 1.636	0.281
Food insecurity	−0.189	−0.031	−0.65	0.516	− 0.76	0.382
Unmet healthcare need	0.792	0.073	1.532	0.126	− 0.224	1.809
WHO-5 Well-being score	0.065	0.053	0.97	0.332	− 0.067	0.197
WC	− 0.076	− 0.147	− 1.113	0.266	− 0.209	0.058
WHR	9.276	0.12	1.704	0.089	− 1.421	19.972
WHtR	9.084	0.118	1.523	0.129	− 2.639	20.807
TBF	0.174	0.245	1.67	0.096	− 0.031	0.38
VFL	− 0.25	− 0.176	− 1.632	0.103	− 0.552	0.051
RM	0.001	0.034	0.32	0.749	− 0.003	0.005
BMI	0.205	0.153	1.181	0.238	− 0.136	0.546
SF	− 0.288	− 0.378	− 2.312	0.021^*^	− 0.534	− 0.043
SM	− 0.121	− 0.105	− 0.704	0.482	− 0.458	0.216
Total PA Level	0	0.145	2.837	0.005^**^	0	0
*R*^2^ (%)	6.400					
Adjusted *R*^2^ (%)	2.700					
DMS score						
Sex	− 6.494	− 0.232	− 2.397	0.017^*^	− 11.819	− 1.169
Ethnicity	1.226	0.079	1.721	0.086	− 0.174	2.625
Financial adequacy	− 1.574	− 0.081	− 1.754	0.08	− 3.338	0.189
Health perception	− 0.05	− 0.003	− 0.053	0.958	− 1.903	1.804
Life perception	− 2.63	− 0.111	− 2.251	0.025^*^	− 4.927	− 0.334
Food insecurity	− 0.673	− 0.043	− 0.968	0.334	− 2.041	0.694
Unmet healthcare need	1.38	0.05	1.114	0.266	− 1.055	3.815
WHO-5 Well-being score	0.238	0.076	1.479	0.14	− 0.078	0.554
WC	− 0.16	− 0.122	− 0.983	0.326	− 0.48	0.16
WHR	13.754	0.069	1.055	0.292	− 11.873	39.382
WHtR	15.992	0.082	1.119	0.264	− 12.095	44.08
TBF	0.158	0.086	0.629	0.529	− 0.334	0.649
VFL	− 0.349	− 0.096	− 0.949	0.343	− 1.071	0.374
RM	0.004	0.072	0.728	0.467	− 0.006	0.013
BMI	0.435	0.127	1.048	0.295	− 0.381	1.252
SF	− 0.359	− 0.184	− 1.2	0.231	− 0.946	0.229
SM	0	0	0	0.999	− 0.808	0.807
Total PA level	0.001	0.124	2.605	0.009^**^	0	0.001
*R*^2^ (%)	17.900					
Adjusted *R*^2^ (%)	14.600					
MDDI score						
Sex	− 2.38	− 0.126	− 1.269	0.205	− 6.067	1.306
Ethnicity	0.364	0.035	0.738	0.461	− 0.605	1.333
Financial adequacy	− 1.227	− 0.093	− 1.974	0.049^*^	− 2.448	− 0.006
Health perception	− 0.649	− 0.049	− 0.993	0.321	− 1.932	0.635
Life perception	− 1.72	− 0.108	− 2.127	0.034^*^	− 3.31	− 0.131
Food insecurity	− 0.626	− 0.06	− 1.3	0.194	− 1.573	0.321
Unmet healthcare need	1.392	0.075	1.623	0.105	− 0.294	3.078
WHO-5 Well-being score	0.013	0.006	0.121	0.904	− 0.205	0.232
WC	− 0.086	− 0.097	− 0.762	0.446	− 0.307	0.136
WHR	8.963	0.067	0.993	0.321	− 8.779	26.706
WHtR	19.567	0.148	1.978	0.049^*^	0.122	39.013
TBF	0.081	0.066	0.467	0.641	− 0.26	0.421
VFL	− 0.428	− 0.175	− 1.682	0.093	− 0.928	0.072
RM	0.004	0.108	1.071	0.285	− 0.003	0.01
BMI	0.367	0.159	1.275	0.203	− 0.199	0.932
SF	− 0.132	− 0.1	− 0.638	0.524	− 0.539	0.275
SM	− 0.115	− 0.058	− 0.404	0.686	− 0.674	0.444
Total PA level	0.001	0.172	3.506	0.001^**^	0	0.001
*R*^2^ (%)	13.700					
Adjusted *R*^2^ (%)	10.200					
MOET score						
Sex	− 1.15	− 0.044	− 0.439	0.661	− 6.298	3.999
Ethnicity	0.085	0.006	0.124	0.901	− 1.268	1.439
Financial adequacy	− 2.168	− 0.119	− 2.498	0.013^*^	− 3.873	− 0.462
Health perception	0.071	0.004	0.078	0.938	− 1.721	1.863
Life perception	− 1.781	− 0.081	− 1.577	0.116	− 4.002	0.439
Food insecurity	− 1.402	− 0.097	− 2.083	0.038^*^	− 2.724	− 0.079
Unmet healthcare need	1.544	0.06	1.289	0.198	− 0.81	3.899
WHO-5 Well-being score	0.28	0.095	1.799	0.073	− 0.026	0.585
WC	− 0.264	− 0.215	− 1.679	0.094	− 0.573	0.045
WHR	35.691	0.192	2.831	0.005^**^	10.912	60.47
WHtR	28.718	0.157	2.078	0.038^*^	1.56	55.876
TBF	0.279	0.163	1.152	0.25	− 0.197	0.754
VFL	− 0.562	− 0.166	− 1.582	0.114	− 1.261	0.136
RM	0.003	0.064	0.624	0.533	− 0.006	0.012
BMI	0.747	0.234	1.86	0.064	− 0.042	1.537
SF	− 0.289	− 0.158	− 1	0.318	− 0.857	0.279
SM	− 0.08	− 0.029	− 0.202	0.84	− 0.861	0.7
Total PA level	0	0.1	2.017	0.044^*^	0	0.001
*R*^2^ (%)	12.200					
Adjusted *R*^2^ (%)	8.600					
Weight loss score						
Sex	0.26	0.129	1.322	0.187	− 0.126	0.646
Ethnicity	0.07	0.063	1.355	0.176	− 0.032	0.172
Financial adequacy	− 0.035	− 0.025	− 0.541	0.589	− 0.163	0.093
Health perception	0.024	0.017	0.344	0.731	− 0.111	0.158
Life perception	− 0.116	− 0.068	− 1.367	0.172	− 0.283	0.051
Food insecurity	0.033	0.03	0.66	0.51	− 0.066	0.133
Unmet healthcare need	0.194	0.098	2.16	0.031^*^	0.018	0.371
WHO-5 Well-being score	− 0.02	− 0.087	− 1.692	0.091	− 0.043	0.003
WC	− 0.023	− 0.241	− 1.926	0.055	− 0.046	0
WHR	0.904	0.063	0.955	0.34	− 0.956	2.763
WHtR	3.594	0.255	3.465	0.001^**^	1.555	5.632
TBF	0.006	0.048	0.348	0.728	− 0.029	0.042
VFL	− 0.003	− 0.013	− 0.128	0.898	− 0.056	0.049
RM	0	0.094	0.942	0.347	0	0.001
BMI	0.059	0.238	1.943	0.053	0	0.118
SF	0.001	0.007	0.044	0.965	− 0.042	0.044
SM	0.01	0.049	0.347	0.729	− 0.048	0.069
Total PA level	6.208E−05	0.175	3.629	< 0.001^**^	0	0
*R*^2^ (%)	16.400					
Adjusted *R*^2^ (%)	13.000					

*DLS *Drive for Leanness Scale,* DMS *Drive for Muscularity Scale,* MDDI *Muscle Dysmorphia Disorder Inventory,* MOET *Muscularity-Oriented Eating Test,* WC* waist circumference, *WHR *waist-hip ratio, *WHtR* waist-height ratio, *TBF* total body fat, *VFL* visceral fat level, *RM *resting metabolism, BMI: body mass index, *SF* subcutaneous fat, *SM* skeletal muscle. Multiple linear regression model with MD-related body image scores as the dependent variable. Standardized regression coefficients (β), *t*-values, 95% confidence intervals, *p*-values, and *R*^2^ are reported. *P*-vales are significant at *p < 0.05 or **p < 0.01

### Body image concerns across PA categories

When examined by PA category, participants in the high PA group showed higher scores across all MD-related body image concern domains (Table 5). Significant trends were observed for DLS (*p* = 0.011), DMS (*p* < 0.001), MDDI (*p* < 0.001), MOET (*p* < 0.001), and weight loss scores (*p* = 0.001). These findings suggest a dose–response relationship, whereby increasing PA levels are associated with progressively higher levels of muscularity-driven body image concerns and behaviors related to exercise and weight control.

**Table 5:** Differences in muscle dysmorphia-related body image concern scores between physical activity categories

	Low (*n* = 133)	Moderate (*n* = 221)	High (*n* = 159)	*p*
DLS score	20.00 ± 7.00	21.00 ± 7.00	22.00 ± 7.00	0.0011^*^
DMS score	30.00 ± 20.00	34.00 ± 21.00	40.00 ± 20.00	< 0.001^**^
MDDI score	21.00 ± 10.00	23.00 ± 12.00	25.00 ± 13.00	< 0.001^**^
MOET score	18.00 ± 13.00	21.00 ± 17.00	24.00 ± 18.00	< 0.001^**^
Weight loss score	4.00 ± 1.00	5.00 ± 1.00	5.00 ± 2.00	0.001^**^

Values are median ± interquartile range. *p* values by Kruskal–Wallis test, significant at ^*^p < 0.05 or ^**^p < 0.01

## Discussion

This study examined associations between MD–related body image concerns, PA, and body composition among young Asian adults in Malaysia, yielding findings that extend beyond geographic replication of Western research. Although several anthropometric and body composition measures were statistically associated with MD–related concerns, these relationships were weak and accounted for little variance. This pattern indicates that, in this population, MD is only minimally explained by objective physical characteristics, despite measurable differences in muscularity, adiposity, and PA levels. These findings challenge Western-centric models that implicitly position inadequate muscularity or excess fat as central drivers of MD [2, 28]. Instead, they support a conceptualization of MD as a predominantly cognitive–affective and socially mediated body image disturbance, in which subjective self-evaluation outweighs physical embodiment [29]. This interpretation aligns with evidence that individuals with MD frequently exhibit limited objective physical deviation despite marked psychological distress, particularly outside elite athletic or bodybuilding contexts [5]. By demonstrating the limited predictive value of physical variables, this study refines existing theoretical frameworks and highlights their cultural boundary conditions. Practically, the findings caution against reliance on body composition or PA metrics as proxies for muscle dysmorphia risk and underscore the importance of psychologically grounded assessment and intervention approaches, particularly in young adult and university populations.

The sex differences observed in this study align with global trends, where males tend to report higher muscularity-oriented body image concerns, while females are more likely to express dissatisfaction with body fat and weight. The significantly higher DMS and MDDI scores among male participants reflect a strong internalization of the muscular ideal, a body image schema characterized by the belief that one’s body is insufficiently muscular or lean [2, 18]. This internalization is further reinforced by sociocultural influences such as mass media, fitness culture, and peer norms, which often equate masculinity with muscularity and physical dominance [7–9]. In contrast, females in this study reported lower PA levels and exhibited higher levels of TBF and VFL. These gendered differences in body ideals may drive divergent motivations for engaging in PA. While males may be more motivated by goals related to muscular development and physique enhancement, females are often driven by weight loss or general health concerns [30, 31]. Importantly, the higher prevalence of high PA among males in our study may reflect not only health-promoting behaviors but also potential risk for compulsive or dysmorphic patterns of exercise. Research has shown that males who score high on MD scales are more likely to engage in rigid exercise routines, sometimes at the expense of social and occupational functioning [10–12]. This highlights the need to distinguish between health-enhancing versus pathology-driven PA, especially in university environments where appearance pressures are prevalent.

The associations between PA levels and MD-related body image concerns observed in this study underscore the complex psychological motivations behind exercise behaviors in young adults. While regular PA is associated with a range of physical and mental health benefits—including improved mood, stress reduction, and weight management—it may also serve as a coping mechanism for body dissatisfaction or distorted body image, particularly among individuals who perceive themselves as falling short of sociocultural ideals [13]. In our study, higher PA levels were significantly associated with elevated scores on the DMS, MDDI, MOET, and weight loss scales. These findings suggest that exercise in this population, particularly males, may be, at least in part, driven by maladaptive cognitive and emotional processes, such as fear of being inadequately muscular, obsessive control over body shape, or anxiety related to perceived flaws in appearance [13, 32].

This interpretation aligns with the concept of appearance-based exercise motivation, in which PA is pursued not for intrinsic enjoyment or health benefits, but for altering physical appearance [28, 33]. Such motivations have been linked to poorer body image outcomes, lower psychological well-being, and higher risk of developing disordered behaviors like compulsive exercise or eating disorders. In males, high levels of muscularity dissatisfaction have been associated with performance-enhancing substance use, social withdrawal, and low self-esteem, while in females, body dissatisfaction has traditionally manifested as restrictive eating and over-exercising [34]. Our findings add to this literature by demonstrating that even among a non-clinical sample of Malaysian university students, higher PA is not universally protective and may instead be indicative of underlying body image pathology in certain individuals.

The interplay between objective measures of body composition and subjective body image perceptions adds an important dimension to understanding MD and related concerns. In this study, higher BMI and central adiposity measures were associated with increased muscularity-related concerns and weight loss motivation. These findings may reflect a cognitive distortion in which individuals with higher adiposity view themselves as less physically ideal or less socially desirable, contributing to heightened dissatisfaction and compensatory behaviors such as excessive exercise [35]. Notably, BMI and body fat are not always accurate indicators of fitness or body satisfaction; muscular individuals may fall within overweight BMI ranges, which can distort body perception, especially when compounded by sociocultural standards that prize both leanness and muscularity [29].

Interestingly, SF emerged as a negative associated factor of DLS and DMS scores, suggesting that greater SF may attenuate the pursuit of a lean or muscular physique in some individuals. One possible explanation is that individuals with higher fat stores may perceive the gap between their current and ideal body as too large to be realistically attainable, leading to disengagement from appearance-driven goals. This interpretation is coherent with the self-discrepancy theory, which posits that perceived discrepancies between actual and ideal body states can motivate behavior change, but only when the ideal is viewed as achievable; when discrepancies are perceived as insurmountable, motivational disengagement may occur instead [29, 36]. Importantly, this pattern complements the modest explanatory power of the regression models, in which objective physical variables—including BMI, TBF, and waist-based indices—accounted for only a modest fraction of the variance (6% to 18%) in MD–related concerns. Rather than representing a methodological weakness, these low *R*^2^ values provide meaningful conceptual insight, indicating that MD is not strongly determined by physical embodiment. Instead, the findings support a cognitive–affective and socioculturally mediated conceptualization of MD, in which subjective body evaluation, internalized appearance ideals, and self-perception exert greater influence than objective physiological characteristics. From this perspective, the limited predictive value of anthropometric and body composition measures underscores the inadequacy of relying on physical markers alone to identify risk of MD and highlights the need to prioritize psychological and sociocultural determinants in both research and practice. Contemporary sociocultural models suggest that constructs such as social media internalisation, peer-group appearance comparisons, perceived normative pressures, and exposure to idealized muscular imagery may play a central role in shaping muscularity-oriented concerns. In particular, the internalisation of digitally mediated body ideals and upward social comparison processes may intensify dissatisfaction even in the absence of objective physical deviation. Incorporating these socio-cognitive variables into future models may better account for the substantial unexplained variance observed in the present study, particularly in Asian contexts where rapid digitalisation and hybridisation of Western and local body standards are reshaping body image experiences.

## Limitations and strengths

Several limitations of this study should be acknowledged. First, the cross-sectional design precludes causal inference regarding the directionality of relationships between body image concerns, PA, and body composition. Second, PA was assessed using the IPAQ-SF, which is known to systematically overestimate total PA, particularly within the vigorous-intensity domain [37]. This measurement bias may have inflated absolute PA values and should be considered when interpreting associations between exercise volume and muscle dysmorphia-related concerns. Importantly, however, the IPAQ-SF remains widely used for ranking individuals by PA level in epidemiological research, and the present analyses focused on relative differences and patterns rather than absolute PA thresholds. Nevertheless, future studies employing objective measures such as accelerometry would allow for more precise quantification of exercise behaviors and their relationship with muscle dysmorphia symptomatology. Third, although BMI emerged as a statistically significant associated factor in some regression models, its interpretive value in the context of MD is limited. BMI does not distinguish between lean mass and adiposity and may therefore misclassify individuals with higher muscularity as overweight, a limitation that is particularly salient in populations with elevated PA or muscularity-oriented concerns [38]. Consequently, associations involving BMI should be interpreted cautiously and in conjunction with more specific body composition indicators. While bioelectrical impedance analysis was used to provide additional estimates of fat and muscle mass, future studies employing more precise techniques, such as dual-energy X-ray absorptiometry, may further clarify physiological correlates. Finally, although validated instruments were used to assess most MD-related constructs, one self-reported measure (Weight Loss Experience Questionnaire) demonstrated only modest internal consistency (McDonald’s ω = 0.55). It indicates a degree of measurement error that may have attenuated observed correlations and regression coefficients. As a result, associations involving this variable may have been underestimated, and conclusions drawn from these findings should be interpreted with caution. The use of an instrument with limited reliability also raises concerns regarding construct precision, as measurement noise may obscure true relationships. Accordingly, findings related to this measure should be considered exploratory, and future research should prioritize the use of more rigorously validated instruments or further psychometric refinement to enhance reliability and construct validity.

Despite these limitations, the study has notable strengths, including a relatively large and ethnically diverse sample, the use of validated psychometric instruments to assess MD-related constructs, and the integration of psychological, behavioral, and body composition measures. Together, these strengths enhance the robustness of the findings and provide a comprehensive examination of MD-related body image concerns in a non-Western young adult population.

## Conclusions

In conclusion, this study provides novel insights into the associations between –related body image concerns, PA, and body composition among university students in Malaysia. Muscularity-driven body image concerns were more pronounced among males and showed statistically significant but modest associations with higher PA levels and selected body composition indicators. Importantly, the limited explanatory power of objective physical measures suggests that psychological and sociocultural factors play a more substantial role in shaping MD-related concerns in this population. These findings underscore the importance of incorporating psychological dimensions into health promotion strategies and support the need for early, gender-sensitive interventions that focus on body image perceptions and exercise motivations rather than physical characteristics alone. From a practical perspective, health and fitness professionals in Malaysia should prioritize screening and intervention approaches that address maladaptive body image cognitions, appearance-driven exercise behaviors, and sociocultural influences such as peer comparison and social media exposure, particularly within university and fitness settings.

## Supplementary Information

Supplementary Material 1: Table S1. STROBE Checklist for Cross-Sectional Studies.

## Data Availability

The datasets used and analysed during the current study are available from the corresponding author on reasonable requests.

## Abbreviations

BMI: Body Mass Index
DLS: Drive for Leanness Scale
DMS: Drive for Muscularity Scale
IPAQ-SF: International Physical Activity Questionnaire–Short Form
IQR: Interquartile range
MDDI: Muscle Dysmorphic Disorder Inventory
MET: Metabolic Equivalent of Task
MOET: Muscularity-Oriented Eating Test
PA: Physical activity
RM: Resting metabolism
SF: Subcutaneous fat
SM: Skeletal muscle mass
SPSS: Statistical Package for the Social Sciences
TBF: Total body fat
VFL: Visceral fat level
WC: Waist circumference
WHO-5: World Health Organization-5 Well-Being Index
WHR: Waist-to-hip ratio
WHtR: Waist-to-height ratio
