# Supplementary material for: Associations between muscle dysmorphia-related body image concerns, physical activity, and body composition: a cross-sectional study among young Asian adults in Malaysia
Source: J Physiol Anthropol. 2026 May 19;45:17. doi: 10.1186/s40101-026-00427-9 (PMC13361597; doi:10.1186/s40101-026-00427-9)
Supplement: Supplementary file 1 — Supplementary Material 1: Table S1. STROBE Checklist for Cross-Sectional Studies. [file 40101_2026_427_MOESM1_ESM.docx]

**Supplementary Table S1**. STROBE Checklist for Cross-Sectional Studies

|  | | | Item No. | Recommendation | Page  No. | | | Relevant text from manuscript |
| --- | --- | --- | --- | --- | --- | --- | --- | --- |
| **Title and abstract** | | | 1 | (*a*) Indicate the study’s design with a commonly used term in the title or the abstract | 1 | | | Associations Between Muscle Dysmorphia-Related Body Image Concerns, Physical Activity, and Body Composition: A Cross-sectional Study among Young Asian Adults in Malaysia |
|  |  |  |  | (*b*) Provide in the abstract an informative and balanced summary of what was done and what was found | 2 | | | Abstract |
| Introduction | | | | | | | |  |
| Background/rationale | | | 2 | Explain the scientific background and rationale for the investigation being reported | 3 | | | Background |
| Objectives | | | 3 | State specific objectives, including any prespecified hypotheses | 4 | | | Background |
| Methods | | | | | | | |  |
| Study design | | | 4 | Present key elements of study design early in the paper | 5 | | | Methods – Study Design and Participants |
| Setting | | | 5 | Describe the setting, locations, and relevant dates, including periods of recruitment, exposure, follow-up, and data collection | 5 | | | Methods – Study Design and Participants |
| Participants | | | 6 | (*a*) *Cross-sectional study*—Give the eligibility criteria, and the sources and methods of selection of participants | 5 | | | Methods – Study Design and Participants |
| Variables | | | 7 | Clearly define all outcomes, exposures, predictors, potential confounders, and effect modifiers. Give diagnostic criteria, if applicable | 5 - 8 | | | Methods – Measures |
| Data sources/ measurement | | | 8* | For each variable of interest, give sources of data and details of methods of assessment (measurement). Describe comparability of assessment methods if there is more than one group | 5 - 8 | | | Methods – Measures |
| Bias | | | 9 | Describe any efforts to address potential sources of bias | 8;  16 | | | Methods – Statistical Analysis; Discussion - Limitations and Strengths |
| Study size | | | 10 | Explain how the study size was arrived at | 5 | | | Methods – Study Design and Participants |
| Quantitative variables | | 11 | | Explain how quantitative variables were handled in the analyses. If applicable, describe which groupings were chosen and why | 8 | | Methods – Statistical Analysis | |
| Statistical methods | | 12 | | (*a*) Describe all statistical methods, including those used to control for confounding | 8 | | Methods – Statistical Analysis | |
|  |  |  |  | (*b*) Describe any methods used to examine subgroups and interactions | 8 | | Methods – Statistical Analysis | |
|  |  |  |  | (*c*) Explain how missing data were addressed | 8 | | Methods – Statistical Analysis | |
|  |  |  |  | (*d*) *Cross-sectional study*—If applicable, describe analytical methods taking account of sampling strategy | Not applicable | | Convenience sampling | |
|  |  |  |  | (*e*) Describe any sensitivity analyses | 8 | | Methods – Statistical Analysis | |
| Results | | | | | | | | |
| Participants | | 13* | | (a) Report numbers of individuals at each stage of study—eg numbers potentially eligible, examined for eligibility, confirmed eligible, included in the study, completing follow-up, and analysed | 5 | | Methods – Study Design and Participants | |
|  |  |  |  | (b) Give reasons for non-participation at each stage | Not applicable | | All questions in the Google Form were set as compulsory, and full completion is required before clicking the “submit” button. Therefore, the participation rate was considered as 100%. | |
|  |  |  |  | (c) Consider use of a flow diagram | Not applicable | |  | |
| Descriptive data | | 14* | | (a) Give characteristics of study participants (eg demographic, clinical, social) and information on exposures and potential confounders | 10 | | Results - Sociodemographic and Lifestyle Characteristics; Table 1 | |
|  |  |  |  | (b) Indicate number of participants with missing data for each variable of interest | Not applicable | |  | |
| Outcome data | | 15* | | *Cross-sectional study—*Report numbers of outcome events or summary measures | 10 - 13 | | Results; Tables 2 – 5 | |
| Main results | | 16 | | (*a*) Give unadjusted estimates and, if applicable, confounder-adjusted estimates and their precision (eg, 95% confidence interval). Make clear which confounders were adjusted for and why they were included | 11 – 12 | | Results; Table 3 - 4 | |
|  |  |  |  | (*b*) Report category boundaries when continuous variables were categorized | 11 – 12 | | Results; Tables 3 – 4 | |
|  |  |  |  | (*c*) If relevant, consider translating estimates of relative risk into absolute risk for a meaningful time period | Not applicable | |  | |
| Other analyses | 17 | | Report other analyses done—eg analyses of subgroups and interactions, and sensitivity analyses | | 11 – 12 | Results; Tables 3 – 4 | | |
| Discussion | | | | | | | | |
| Key results | 18 | | Summarise key results with reference to study objectives | | 13 | Discussion – opening paragraph | | |
| Limitations | 19 | | Discuss limitations of the study, taking into account sources of potential bias or imprecision. Discuss both direction and magnitude of any potential bias | | 17 | Discussion – Limitations and Strengths | | |
| Interpretation | 20 | | Give a cautious overall interpretation of results considering objectives, limitations, multiplicity of analyses, results from similar studies, and other relevant evidence | | 17 | Discussion – Limitations and Strengths | | |
| Generalisability | 21 | | Discuss the generalisability (external validity) of the study results | | 17 | Discussion – Limitations and Strengths | | |
| Other information | | |  | | | | | |
| Funding | 22 | | Give the source of funding and the role of the funders for the present study and, if applicable, for the original study on which the present article is based | | 19 | Declarations – Funding | | |
